# Patient-derived stem cell models of brain endothelial-neuron interactions in early course psychosis

**DOI:** 10.3389/fpsyt.2026.1873300

**Published:** 2026-09-29

**Authors:** Deniz Altunsu, Cemal Demirlek, Jennifer Nicholson, Brendan Stiltner, Rebekah Trotti, Nicolas Raymond, Yukyeong Lee, Rufuto Rahman, William Stone, Rakesh Karmacharya, Paulo Lizano

**Affiliations:** 1 Department of Psychiatry, University of Rochester Medical Center, Rochester, NY, United States; 2 Department of Psychiatry, Beth Israel Deaconess Medical Center, Boston, MA, United States; 3 Department of Psychiatry, Harvard Medical School, Boston, MA, United States; 4 Center for Genomic Medicine, Massachusetts General Hospital, Boston, MA, United States; 5 Chemical Biology Program, Broad Institute, Cambridge, MA, United States; 6 Schizophrenia and Bipolar Disorder Program, McLean Hospital, Belmont, MA, United States

**Keywords:** blood-brain barrier, brain microvascular endothelial cell (BMEC), early course psychosis, iPSC (induced pluripotent stem cell), neurovascular unit (NVU)

## Abstract

**Introduction:**

Early-course psychosis (ECP) exhibits marked heterogeneity in illness trajectories, with a substantial subset of individuals developing progressive symptomatic, cognitive, and functional decline. Although growing evidence implicates blood-brain barrier (BBB) and neurovascular unit (NVU) dysfunction in psychosis spectrum disorders, direct links between longitudinal clinical trajectories and disease-related cellular phenotypes remain insufficiently characterized.

**Methods and analysis:**

This protocol describes a standardized stem cell-based framework to model BBB and NVU dysfunction in ECP using induced pluripotent stem cells (iPSCs) derived from peripheral blood mononuclear cells (PBMCs) within a longitudinally characterized clinical cohort. The approach integrates donor-specific *ex vivo* cellular assays with repeated clinical, cognitive, and functional assessments for up to 5 years. Procedures are detailed for iPSC reprogramming from PBMCs, differentiation of iPSCs to brain microvascular endothelial cells (BMECs) and cortical neurons, and functional assessment of BBB integrity using trans-endothelial electrical resistance (TEER), paracellular permeability, and tight junction protein expression. NVU effects are examined by exposing cortical neurons to BMEC-conditioned media under matched and cross-culture conditions, with quantification of dendritic spine morphology, synaptic puncta, and neuronal activity using micro-electrode arrays. The protocol further incorporates modular mechanistic perturbations, including pharmacological and genetic modulation of matrix metalloproteinase 1 (MMP1), tumor necrosis factor alpha (TNFα)-related signaling, and evaluation of antipsychotic effects on barrier function.

**Ethics and dissemination:**

Ethical approval and informed consent for this study was obtained and the findings will be disseminated through publication in peer-reviewed journals and presentations at scientific conferences.

## Strengths and limitations of this study

- This protocol integrates longitudinal clinical phenotyping with iPSC models of BBB and NVU function.
- The design enables direct linkage between *ex vivo* cellular phenotypes and longitudinal clinical trajectories in ECP.
- The study incorporates multimodal experimental readouts including barrier integrity assays, neuronal morphology, electrophysiology, proteomics, and transcriptomics.
- Mechanistic perturbation strategies using pharmacological inhibition and genetic manipulation enable investigation of causal pathways contributing to barrier dysfunction.
- As an *ex vivo* platform that does not include all features and cell types present in the human brain, the model does not fully recapitulate the complexity of the *in vivo* neurovascular environment.

## Introduction

Early-course psychosis (ECP) is characterized by marked heterogeneity in symptom severity, cognition, and functional outcomes (1). While some individuals achieve sustained remission, approximately 30-40% exhibit a neuroprogressive trajectory marked by persistent symptoms, cognitive decline, and functional deterioration (2–5). Although early intervention services improve short-term outcomes, they have limited impact on long-term illness progression, underscoring the need for mechanistic models that explain neuroprogression in early psychosis. Addressing this gap requires moving beyond symptom-based frameworks to examine the cellular and circuit-level processes that may contribute to illness heterogeneity, including interactions among neuronal and brain microvascular endothelial cells (BMECs) within the neurovascular unit (NVU). Specialized BMECs form the blood brain barrier (BBB), which interacts with neurons, astrocytes, pericytes, and extracellular matrix components within the NVU (6).

Psychosis has traditionally been conceptualized as a disorder of synaptic function with converging genetic, postmortem and imaging evidence implicating alterations in glutamatergic signaling, dendritic spine density and cortical circuit organization (7–9). However, accumulating evidence implicates non-neuronal cell types within the NVU, including BMECs in psychosis pathophysiology (10). Genetic studies demonstrate enrichment of endothelial transport and junctional genes, including solute carrier family 39 member-8 (SLC39A8), low-density lipoprotein receptor-related protein-1 (LRP1), endothelial cell-selective adhesion molecule (ESAM), and claudin-5 (CLDN5) (11–16). Postmortem and transcriptomic studies further reveal endothelial structural abnormalities and dysregulated inflammatory and angiogenic signaling, collectively supporting BBB dysfunction in psychosis (17–20).

Peripheral and *in vivo* biomarkers provide convergent evidence for BBB disruption. Elevated levels of endothelial injury markers (S100B, VEGFA, and ICAM1) have been reported in blood and cerebrospinal fluid (CSF) in ECP and chronic schizophrenia (21–24). Increased immune cell and immunoglobulin extravasation, elevated CSF-to-serum albumin ratios, and increased circulating brain-derived cell-free DNA fragments further support compromised barrier integrity (21, 25–29). Importantly, dynamic contrast-enhanced MRI (DCE-MRI) studies demonstrate pathological BBB leakage in subsets of individuals with bipolar disorder and ECP, with greater permeability associated with symptom severity, illness duration, cardiometabolic risk, and neuroprogression (30–32). Together, these findings suggest that BBB disruption may represent a biologically meaningful and measurable substrate of illness heterogeneity. A deeper understanding of BBB and NVU biology may therefore inform the development of predictive biomarkers capable of stratifying patients, forecasting clinical trajectories and potentially monitoring treatment response in psychosis.

Despite recent advances, mechanistic understanding of BBB dysfunction in psychosis remains limited. Most studies rely on postmortem tissue, animal models, or indirect imaging approaches that do not permit controlled investigation of patient-specific BMEC phenotypes. Moreover, clinical and cellular studies are typically conducted in separate cohorts, precluding direct linkage between *ex vivo* cellular abnormalities and longitudinal clinical trajectories within the same individuals. Induced pluripotent stem cell (iPSC) technology provides a patient-specific and scalable platform to address these limitations (33–36). Advances in reprogramming and differentiation enable generation of BMECs and cortical neurons from peripheral blood mononuclear cells, preserving donor genetic background while allowing standardized functional assays (37–39). iPSC-derived BBB models recapitulate key BMEC properties, including expression of brain endothelial markers, high trans-endothelial electrical resistance, selective efflux transporter activity, and angiogenic capacity (40–42). A limited number of studies using iPSC-derived BMECs in psychosis demonstrate reduced barrier integrity, increased permeability, altered immune signaling, and transcriptional dysregulation of junctional and angiogenic pathways, supporting a primary role for BMEC dysfunction (42–44).

Parallel progress in iPSC-derived neuronal models has provided insight into synaptic and circuit-level abnormalities in psychosis (45–47). Genetic and postmortem studies converge on synaptic pathology, particularly reductions in dendritic spine density and altered pyramidal neuron morphology (48–50). iPSC-derived cortical neurons reproduce disease-relevant alterations in dendritic architecture, synaptic organization, mitochondrial function, and electrophysiological activity (51–53). However, neuronal phenotypes are increasingly recognized as being shaped by extrinsic influences arising from endothelial and immune signaling within the NVU (54).

Mechanistic dissection of BBB dysfunction requires experimental systems that allow targeted perturbation of candidate pathways. Inflammatory signaling and matrix metalloproteinases, particularly matrix metalloproteinase 1 (MMP1), regulated by tumor necrosis factor alpha (TNFα) signaling, have emerged as key mediators of barrier disruption through degradation of extracellular matrix and tight junction proteins (55–57). Rho-associated kinase signaling further regulates tight junction (claudin-5, occludin) phosphorylation and localization (58). Pharmacological and genetic modulation of these pathways improves barrier integrity *in vitro*, highlighting their relevance as mechanistic targets (42). Emerging evidence also suggests that antipsychotic medications may differentially modulate BBB function, although systematic evaluation remains limited (17).

The present prospective study protocol addresses these gaps by integrating longitudinal clinical phenotyping with patient-specific iPSC-derived models of BBB and NVU function. Standardized assays of endothelial integrity are combined with transcriptomic profiling, mechanistic perturbation strategies, and endothelial-neuronal interaction paradigms. By anchoring *ex vivo* cellular phenotypes to longitudinal clinical trajectories within the same individuals, this approach provides a translational platform to investigate BMEC contributions to neuroprogression in early psychosis and to support biologically informed stratification and therapeutic discovery.

## Methods and analysis

### Study objectives and aims

The following framework combines repeated assessments of symptoms, cognition, and functional outcomes with complementary functional, molecular, and transcriptomic characterizations of iPSC-derived BMECs, and cortical neurons generated from PBMC obtained from individuals with ECP and HCs. Within this framework, the study is designed to (Aim 1) characterize *ex vivo* BBB integrity in relation to longitudinal markers of neuroprogression in ECP, (Aim 2) model NVU dysfunction by systematically examining interactions between iPSC-derived BMECs and cortical neurons, and (Aim 3) define molecular mechanisms underlying BBB impairment through targeted pharmacological and genetic perturbations.

This manuscript describes a prospective study protocol. It reports the planned study design and standardized experimental procedures for the future study. No human participant data are reported in the present manuscript (see Ethics and Dissemination).

### Primary and secondary endpoints

For Aim 1, the primary endpoint is *ex vivo* BBB integrity, quantified as peak impedance-based barrier resistance (TEER) in iPSC-derived BMECs (ECP vs. HC); secondary endpoints are paracellular permeability to fluorescent dextrans (0.1–70 kDa), tight junction protein expression (CLDN5, OCLN, and TJP1 at the mRNA and protein levels), BMEC transcriptomic differentially expressed genes, and the association of these BBB measures with baseline and longitudinal clinical, cognitive, and functional outcomes. For Aim 2, the primary endpoint is dendrite length in cortical neurons exposed to BMEC-conditioned media, with spontaneous spike frequency on micro-electrode arrays as a co-primary functional readout; secondary endpoints are dendritic spine density, synaptic puncta density (Homer/Bassoon co-localization), and neuronal transcriptomic change, evaluated across matched and cross-culture (ECP/HC) conditions. For Aim 3, the primary endpoint is rescue of barrier integrity following pharmacological or genetic perturbation of MMP1, with the converse impairment expected upon MMP1 overexpression; secondary endpoints are paracellular permeability, tight junction protein expression and Rho-kinase-dependent phosphorylation (CLDN5 T207; OCLN T382/S507), perturbation-associated transcriptomic signatures, downstream neuronal morphology and activity in BMEC-neuron assays, and the comparative effects of antipsychotics on barrier function.

### Participants and blood collection

participants will include 40 individuals with ECP and 20 healthy controls (HCs) that are balanced for sex, race, and age. They will be recruited at Beth Israel Deaconess Medical Center, where the Institutional Review Board has approved the study and all subjects will provide written informed consent.

### Sample size and feasibility

The proposed sample of 40 ECP participants and 20 HCs (matched for sex, race, and age; 18–45 years) exceed those of comparable iPSC studies of BBB and neuronal phenotypes in psychosis, which detected group differences with 8–14 lines (42, 43, 52). Because the assay panel is extensive, experiments are tiered. All 60 donors undergo the core Aim 1 pipeline: reprogramming, BMEC differentiation, impedance-based barrier integrity (TEER), permeability, tight junction protein expression, and RNA sequencing. Resource-intensive or confirmatory assays are performed in pre-specified subsets balanced for diagnosis, sex, and age: efflux transporter validation (3 HC, 3 ECP lines); the Aim 2 neuron and cross-culture experiments (differentiation, conditioned-media exposure, imaging, MEA, RNA-seq) and secretome profiling; and the Aim 3 perturbations in all ECP lines (pharmacological, genetic, and antipsychotic). Because barrier identity and transporter function are reproducible across independent lines and batches (59, 60) and TEER is highly reliable in our pilot data (42), confirmatory validation in a small subset is sufficient.

Based on G*Power analysis (61, 62), at the proposed sample size, the design provides at least 80% power (two-sided α = 0.05) to detect a between-group standardized difference of d ≈ 0.78 for the primary Aim 1 contrast (40 ECP vs. 20 HC) and, within the 40 ECP participants, to detect correlations between barrier integrity and clinical measures of r ≈ 0.43 (r ≈ 0.49 after Bonferroni correction across the three clinical domains; α = 0.017). The study is thus adequately powered to detect even moderate effects. Our preliminary data suggest substantially larger effects: our pilot iPSC-derived BMEC TEER data (d = 1.5), together with a conservative estimate (d = 1.1), yield greater than 95% power for the Aim 1 primary outcome, and TEER-clinical correlations of r = 0.6-0.7 with psychosis symptoms and r = -0.6 to -0.9 with cognition reported in comparable *ex vivo* iPSC studies yield greater than 95% power at r = 0.6 (Bonferroni-corrected across the three domains; p = 0.017). For Aim 2, pilot differences in spontaneous spike frequency (d = 3.5) and dendrite length (d = 5.0) between ECP and HC neurons provide greater than 95% power (two-tailed α = 0.025, 2:1 allocation, Bonferroni-corrected across the two co-primary endpoints). Because the pilot effect sizes are derived from small cohorts and are therefore uncertain, the sample-size justification is anchored on the conservative minimum-detectable effects above, and all power estimates will be refined as effect sizes from initial differentiations accrue.

### Inclusion and exclusion criteria

Participants in the ECP group will meet the following criteria: 1) Age 18–45 years at time of enrollment, 2) DSM-5 diagnosis of schizophrenia (SZ), schizoaffective disorder (SZA), or bipolar I disorder with psychotic features (BDP), or other psychosis spectrum disorder, confirmed by the Structured Clinical Interview for DSM-5 (SCID), with less than 5 years since psychosis onset, 3) English-speaking, 4) Ability to complete the Brief Assessment of Cognition App (BAC App) at baseline, 5) Completion of the 2-subtest version of the Wechsler Abbreviated Scale of Intelligence, Second Edition (WASI-II), to estimate IQ, 6) Mild to moderate psychosis severity, defined by a Positive and Negative Syndrome Scale (PANSS) total score of 58-75, 7) Stable outpatient treatment with no psychiatric hospitalization within the past 30 days, 8) No changes in antipsychotic medication within the past month, or, for individuals receiving long-acting injectable antipsychotics, no changes within the past 2 months. Participants in the HC group will meet the following criteria: 1) No lifetime history of psychotic disorder or major mood disorder, as determined by the SCID (non-patient edition), 2) No family history of psychosis, 3) No current or prior treatment with medications known to affect cognition.

The following exclusion criteria will apply to all participants: 1) Score on the WRAT 4 Word Reading test < 60, or if they speak English as a second language, a WASI matrix reasoning score < 60, 2) Alcohol or substance use disorder within the past 3 months or a positive urine drug screen, 3) History of traumatic brain injury associated with loss of consciousness greater than 15 minutes, 4) History or current major neurological or cognitive disorder that is severe, of long duration, and/or causes functional impairment, 5) History or current major medical disorder that could affect the central nervous system functioning which is severe, of long duration, and/or causes functional impairment, or 6) a positive pregnancy test.

### Clinical and cognitive assessments

Clinical and cognitive data will be collected at baseline and annually for up to 5 years from individuals with ECP and HCs. Clinical assessments will include the SCID, psychiatric, medical, and substance use history, including any family history of psychiatric illness, the Modified Cigarette/E-Cigarette Evaluation Questionnaire (MCECEQ), the Pittsburgh Sleep Quality Index (PSQI), the Clinical Anxiety Scale (CAS), the Birchwood Social Functioning Scale (SFS), the Global Assessment of Functioning (GAF). Among individuals with ECP, the Positive and Negative Syndrome Scale (PANSS), the Young Mania Rating Scale (YMRS), the Montgomery-Åsberg Depression Rating Scale (MADRS), Clinical Global Impressions (CGI) scale, and medication data will also be collected. All participants will also complete self-reported questionnaires, including the Audio-visual abnormalities questionnaire (AVAQ), the Bonn Visual scale, the World Health Organization Disability Assessment Schedule (WHODAS 2.0), the Patient Health Questionnaire-9 (PHQ-9), the General Anxiety Disorder-7 (GAD-7), and the DSM-5 Level 1 Cross-Cutting Symptom Measure-Adult Report. Cognitive performance will be assessed using the WASI-II, providing an estimate of IQ, and the BAC App, which evaluates six cognitive domains: verbal memory, working memory, motor speed, verbal fluency, attention and processing speed, and executive function. The BAC App also generates a composite score of cognitive performance based on the six tasks.

### Generation of iPSCs and differentiation to BMECs

Human iPSC lines will be reprogrammed from peripheral blood mononuclear cells (PBMCs) using a non-integrating Sendai virus reprogramming system (CytoTune) at the Harvard Stem Cell Institute (HSCI), following standardized core facility protocols. For each donor, three independent iPSC clones will be expanded and cryopreserved to establish a cell bank. Each clone will be banked in multiple early-passage aliquots to ensure long-term availability and to minimize passage-related variability across experimental batches. Quality control procedures will include confirmation of pluripotency markers (alkaline phosphatase, SOX2, NANOG), assessment of trilineage differentiation potential, qPCR-based karyotype screening for chromosomal abnormalities, and routine mycoplasma testing. Clones meeting all quality control criteria will be retained, and at least one validated clone per donor will be used for downstream differentiation. Independent differentiations will be performed in biological triplicate to assess reproducibility and between batch reliability. For the main cohort-level analyses, a single validated clone per donor will be used to keep the full 40-ECP/20-HC design tractable. To evaluate clone-level variability, at least two independent clones will be differentiated in a pre-specified subset of donors, and clone will be modeled as a random effect nested within donor in these analyses. Residual clone-specific variability inherent to single-clone designs is acknowledged as a limitation and is addressed through the early-passage banking and multi-clone validation described above.

Patient-derived iPSCs will be cultured on Matrigel in mTeSR+ medium until they reach approximately 80% confluency. Endothelial differentiation will follow the ETS transcription factor-based protocol for its ability to induce a canonical endothelial transcriptional program (63). In contrast to earlier methods that generate epithelial-like cells lacking key endothelial markers (CDH5, PECAM1, KDR), this approach employs lentiviral overexpression of ETV2, ERG, and FLI1 to promote acquisition of brain endothelial identity and robust barrier function.

In **Figure 1**, to differentiate iPSCs to BMECs, confluent iPSC cultures will be transitioned to an unconditioned medium lacking basic fibroblast growth factor (bFGF) for 6 days. On day 6, lentiviral vectors encoding ETV2, ERG, and FLI1 will be introduced, followed by transfer to endothelial cell medium. On day 8, cells will be dissociated with Accutase and seeded onto collagen IV- and fibronectin-coated plates and cultured for 4 days. On day 12, we will obtain the only BMECs expressing the endothelial markers CD31 (PECAM1) and CD144 (VE-cadherin) while excluding cells positive for the epithelial marker CD326 (EPCAM) by performing fluorescence-activated cell sorting. Isolated cells will be seeded onto 0.4-µm Transwell inserts for downstream functional assays. CD326 (EPCAM) is included specifically as a negative selection marker to deplete epithelial-like cells, addressing the recognized concern that some iPSC-derived BBB protocols generate epithelial rather than endothelial cells. Brain endothelial identity of the sorted population will be confirmed by co-expression of canonical endothelial markers (PECAM1/CD31 and CDH5/CD144), functional angiogenic (tube-formation) capacity, and the absence of epithelial markers (EPCAM/CD326), thereby excluding epithelial cells prior to downstream assays.

**Figure 1:**
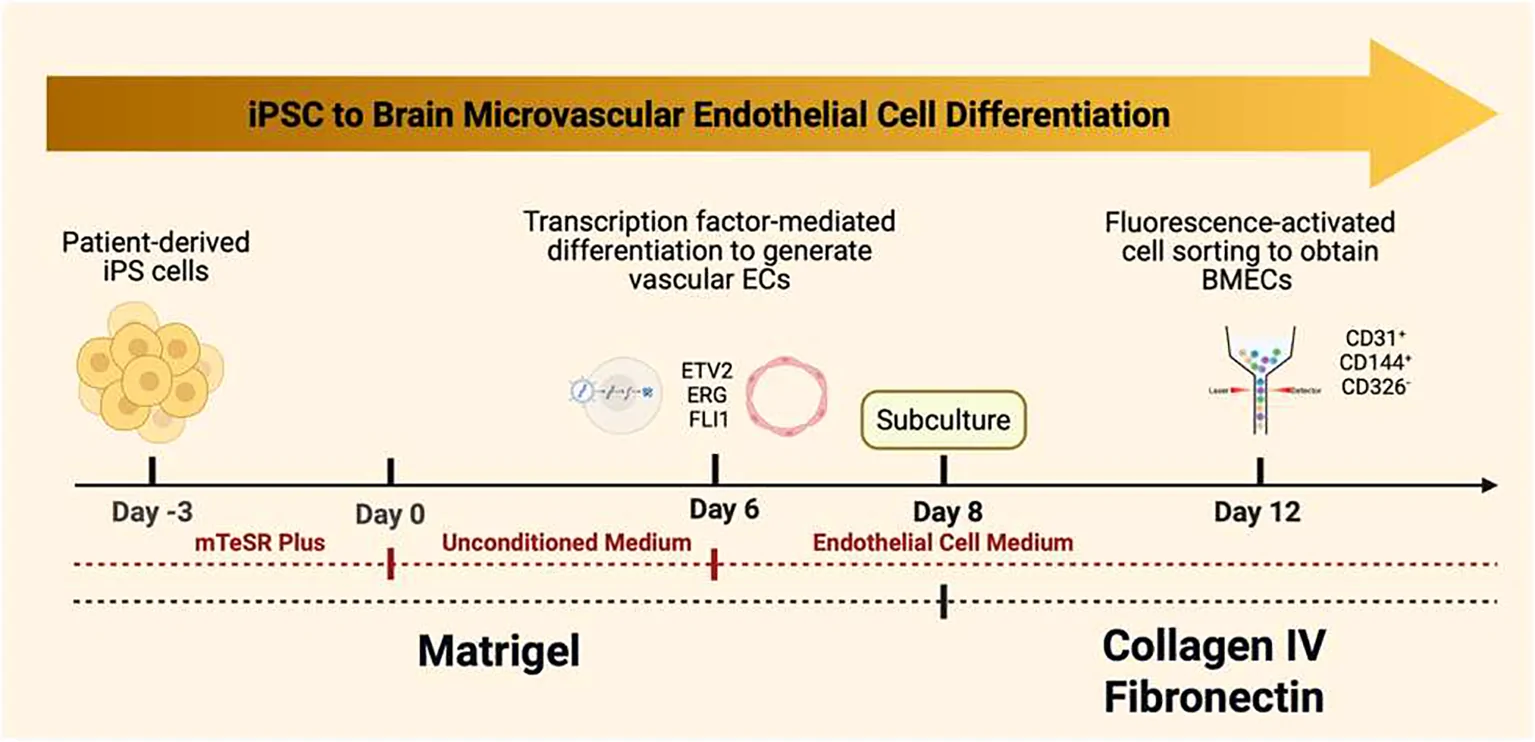
Overview of differentiation of patient-derived induced pluripotent stem cells to brain microvascular endothelial cells. Patient-derived iPSCs are cultured on Matrigel and differentiated into BMECs using lentiviral induction of ETV2, ERG, and FLI1. Cells are subsequently transferred to endothelialmedium and puri ed by uorescence-activated cell sorting to isolate BMECs positive for the endothelial markers CD31 and CD144 and negative for the epithelial marker CD326 (EPCAM). Adapted from Lu et al. (63), Proceedings of the National Academy of Sciences, 118(8), e2016950118 (https://doi.org/10.1073/pnas.2016950118), licensed under CC BY 4.0 (https://creativecommons.org/licenses/by/4.0/).

### Virus production

Lentiviral particles encoding human-ETV2, -FLI1, and -ERG will be generated and concentrated following the protocol (63) with minor modifications, using plasmids encoding ETV2, FLI1 and ERG provided by Dr. Lu and his team. Subconfluent Lenti-X 293T cells will be transfected using the Lenti-X Single-Shot Packaging System according to the manufacturer’s instructions. Viral supernatants will be clarified through a 0.45-μm PES filter and concentrated using Lenti-X Concentrator. The resulting viral pellet will be resuspended in 100 μL lentivirus storage buffer per 100-mm plate of producer cells and stored at -80 °C for long-term use.

### BMEC characterization and functional validation

Tight junction integrity will be evaluated in iPSC-derived BMECs by immunocytochemistry, qPCR and immunoblotting (**Figure 2A**). Cells will be labeled with antibodies against canonical BBB-associated proteins, including claudin-5 (CLDN5), occludin (OCLN), ZO-1 (TJP1), and VE-cadherin (CDH5). Fluorescent images will be acquired under standardized conditions, and junctional continuity and localization will be quantified using automated image analysis pipelines. Protein expression levels will be further assessed by immunoblotting to quantify total and, where applicable, phosphorylated forms of tight junction proteins, with densitometric values normalized to loading controls.

**Figure 2:**
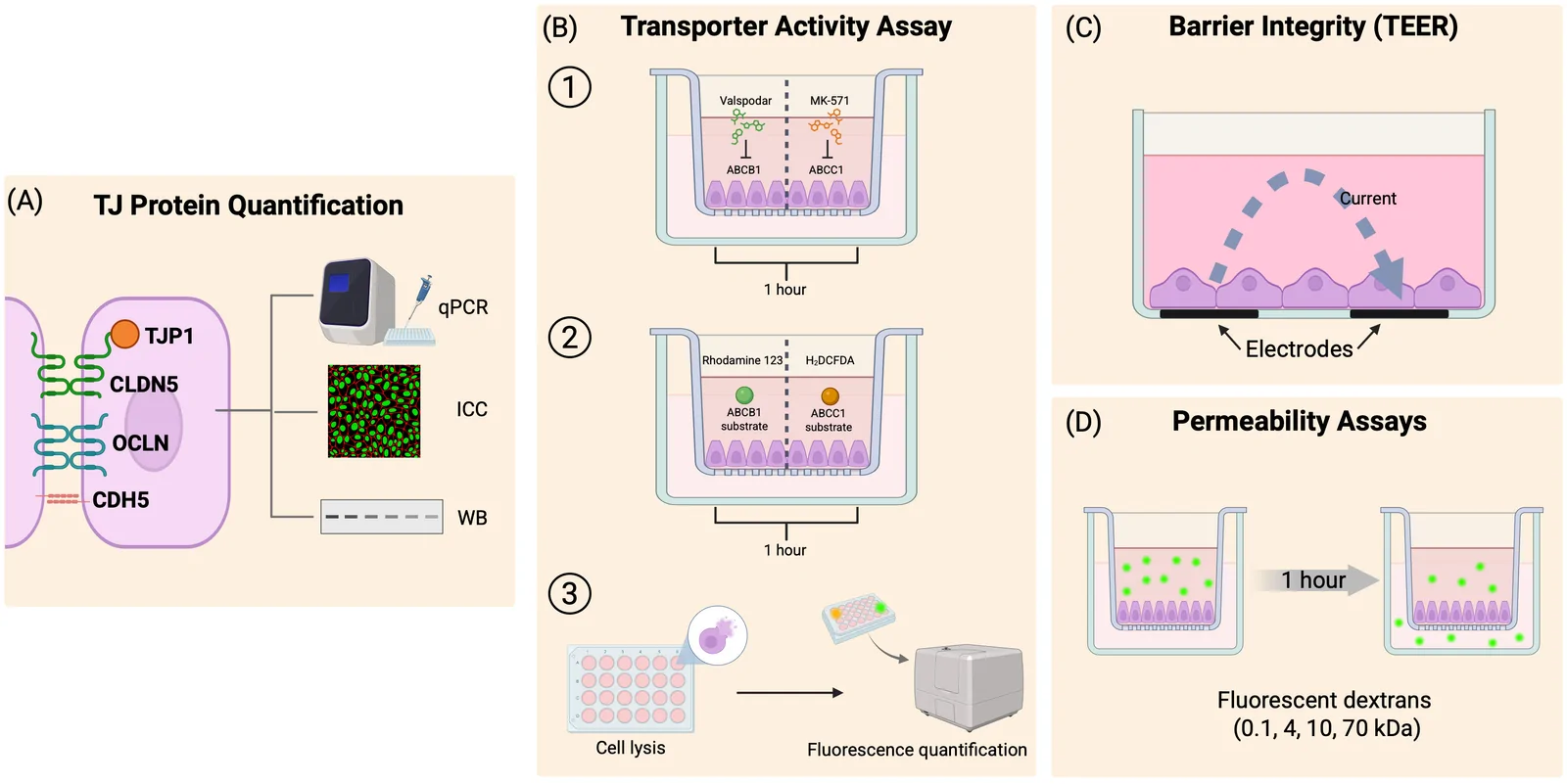
Brain microvascular endothelial cell characterization and barrier integrity assessment. **(A)** Expression of tight junction proteins (claudin-5, occludin, ZO-1, and VE-cadherin) is assessed using immunocytochemistry, immunoblotting, and qPCR. **(B)** Efflux transporter activity (ABCB1 and ABCC1) is evaluated using substrate accumulation assays. **(C)** Barrier integrity is monitored using impedance-based measurements. **(D)** Permeability is assessed by measuring the passage of fluorescent dextrans across endothelial monolayers in a transwell system.

To verify functional brain endothelial identity, a random subset of iPSC-derived BMEC lines (3 HC and 3 ECP) will undergo expanded validation of efflux transporter activity, barrier integrity, and permeability. Efflux transporter function (ABCB1 and ABCC1) will be assessed using substrate accumulation assays, in which cells will be pre-incubated with valspodar (ABCB1 inhibitor) or MK-571 (ABCC1 inhibitor), followed by exposure to rhodamine 123 or H_2_DCFDA, respectively (**Figure 2B**). Intracellular fluorescence will be quantified as a measure of transporter activity.

Validation experiments will be performed in biological triplicate from independent differentiations and in technical triplicate within each experiment to assess within-experiment reliability and between-batch reproducibility. Similar replication strategies will be applied to impedance-based barrier measurements and permeability assays, which represent the primary functional readouts. These validation studies are designed to confirm endothelial identity and functional integrity while avoiding unnecessary transporter profiling across the full cohort. Differentiations will be considered to have failed and will be excluded from analysis if they do not meet pre-specified release criteria, including endothelial purity (e.g., ≥80-90% CD31+/CD144+ and <5% CD326+ by flow cytometry) and characteristic cobblestone endothelial morphology.

### Barrier integrity assays

Endothelial barrier integrity will be assessed continuously using impedance-based measurements on the Axion Maestro Edge platform, which enables continuous, label-free monitoring of barrier formation and stability (**Figure 2C**). iPSC-derived BMECs will be plated on Axion-compatible electrode arrays, and impedance measurements will be recorded longitudinally to quantify barrier development and integrity. Measurements will be acquired at regular intervals under controlled temperature and CO_2_ conditions, enabling real-time assessment of barrier maturation and dynamic responses to experimental perturbations. Compared to traditional chopstick-based TEER systems, impedance-based measurements reduce operator-dependent variability, eliminate manual electrode placement, and allow simultaneous high-throughput monitoring across multiple wells. Impedance values will be normalized to cell-free electrode arrays, corrected for electrode area, and reported as area-corrected resistance (Ω x cm²); measurements will be acquired at regular intervals (approximately every 1 h) from seeding through barrier maturation (typically days 1–6 post-seeding) to enable comparisons across diagnostic groups and experimental conditions.

To assess BBB permeability, iPSC-derived BMECs will be cultured as confluent monolayers on 0.4-µm pore Transwell inserts (**Figure 2D**). Once barrier formation has been confirmed by predefined impedance/TEER criteria, fluorescent dextran tracers of increasing molecular weight (0.1, 4, 10, and 70 kDa) will be added to the apical chamber. Following a 1 h incubation, medium from the basolateral chamber will be collected, and fluorescence intensity will be quantified using a microplate reader. Dextran concentrations will be determined from tracer-specific standard curves, enabling calculation of the apparent permeability coefficient (Papp, cm/s), calculated as Papp = (dQ/dt)/(A × C0), where dQ/dt is the steady-state rate of tracer appearance in the basolateral chamber, A is the membrane surface area, and C0 is the initial tracer concentration in the apical chamber. Increased Papp values will be interpreted as evidence of impaired barrier integrity and increased paracellular permeability. Size selectivity will be evaluated across the dextran molecular-weight series, and replicate measurements from each differentiation will be averaged within donor before donor-level values are used for statistical comparisons.

### Differentiation of iPSCs into cortical neurons

iPSCs derived from ECP and HC lines will be differentiated into cortical neurons using a protocol based on a previously described dual-SMAD inhibition protocol for neural induction of human iPSCs (64), followed by an established cortical neuronal differentiation and maturation protocol (**Figure 3A**). Neural induction will be initiated using dual-SMAD inhibitors to promote neuroectoderm specification, followed by progression through the neural progenitor stage. On day 20 of differentiation, differentiating cells will be dissociated with Accutase and replated onto poly-L-ornithine and laminin-coated plates in Neurobasal A medium supplemented with GlutaMAX and B27. For the first seven days following replating, the Notch inhibitor DAPT will be added to promote neuronal differentiation (65). Long-term neuronal cultures will be maintained with brain-derived neurotrophic factor (BDNF), glial cell line-derived neurotrophic factor (GDNF), dibutyryl cAMP, and ascorbic acid to support neuronal maturation and survival. Neuronal cultures will be maintained under these culture conditions until day 40 of differentiation to allow sufficient maturation prior to analysis and exposure to BMEC-conditioned media.

**Figure 3:**
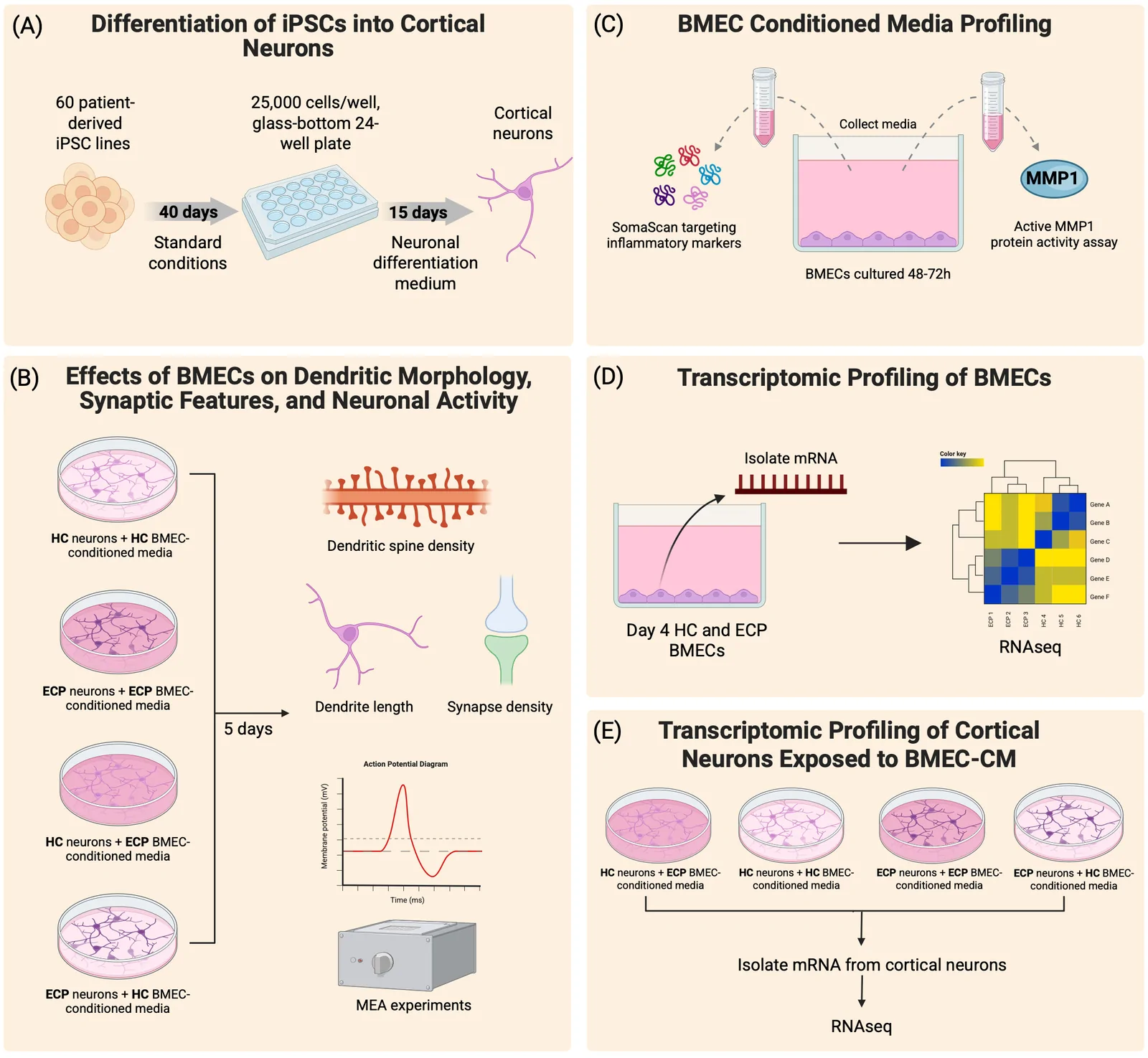
BMEC profiling and interrogation of BMEC-neuron interactions. **(A)** Patient-derived iPSCs are differentiated into cortical neurons using a dual SMAD inhibition protocol. **(B)** Cortical neurons derived from healthy controls (HC) and early-course psychosis (ECP) are exposed to matched and cross-group BMEC-conditioned media, followed by assessment of neuronal morphology and activity. **(C)** BMEC-conditioned media is profiled using SomaScan proteomics and MMP1 ELISA.**(D)** BMECs undergo transcriptomic profiling to identify differentially expressed genes.**(E)** RNA sequencing is performed on neurons following exposure to BMEC-conditioned media to evaluate transcriptional changes.

### Preparation of BMEC-conditioned media

BMEC-derived from ECP and HC iPSC lines will be subcultured under standard differentiation conditions. Cells will be cultured to ~70-90% confluence, washed twice with phosphate-buffered saline to remove residual serum components, and incubated in BMEC media for 48–72 h to allow accumulation of secreted factors. The CM will be harvested and sequentially centrifuged at 300 × g for 10 min and 2,000 × g for 20 min to remove cells and cellular debris. The clarified supernatant will be passed through a 0.22 μm sterile filter to eliminate remaining particulates. Filtered CM will be aliquoted and stored at -80 °C until use. Freeze-thaw cycles will be minimized to preserve the stability of secreted proteins and extracellular vesicles.

### Exposure of cortical neurons to BMEC-CM

On day 40 of neuronal differentiation, cortical neurons will be exposed to BMEC-CM (**Figure 3B**). Neuronal cultures will be maintained for 5 days in a 1:1 mixture of BMEC-CM and neuronal differentiation medium. Matching conditions will include HC neurons exposed to HC BMEC-CM and ECP neurons exposed to ECP BMEC-CM. Cross-culture conditions will include HC neurons exposed to ECP BMEC-CM and ECP neurons exposed to HC BMEC-CM.

### Quantification of dendritic morphology and synaptic features

Following exposure to BMEC-CM, neuronal cultures will be fixed (n>3 independent cultures per condition) and immunolabeled with markers of cortical neuron subtypes, including SATB2 (upper cortical layer neurons) and CTIP2 (deep cortical layer neurons). Dendrites and synapses attached to neuronal cell bodies expressing these markers will be selected for analysis. Images will be acquired using an Opera Phenix high-content imaging system at 63× magnification. Dendritic length and dendritic spine density will be quantified using an automated image analysis pipeline implemented in Harmony high-content analysis software, applying the SER (saddle-edges-ridges) method to outline dendrites and spines. Synaptic puncta will be identified by filtering images for puncta positive for both Homer (postsynaptic marker) and Bassoon (presynaptic marker). Quantification will be performed to derive measures of dendritic length, dendritic spine density, and synaptic puncta density in SATB2-positive and CTIP2-positive neuronal subpopulations (**Figure 3B**).

### Measurement of neuronal activity using micro-electrode arrays

In **Figure 3B**, neuronal activity will be assessed using micro-electrode arrays (MEAs) to record extracellular action potential waveforms in cortical neurons exposed to BMEC-CM, as described previously (46). Spontaneous action potential spikes will be recorded in a real-time and label-free manner. Spike frequency will be quantified as a measure of neuronal activity. Matching and cross-culture conditions will be analyzed to compare neuronal activity in HC and ECP neurons exposed to BMEC-CM derived from the same or the opposite group.

### Profiling of BMEC-CM

CM collected from BMEC cultures will be profiled using the SomaScan proteomic platform (SomaLogic), enabling multiplexed quantification of approximately 300–500 proteins, including cytokines, chemokines, and extracellular matrix regulators (**Figure 3C**). Samples will be assigned randomized identifiers and processed in a blinded manner with respect to the diagnostic group. Data quality control, normalization, and calibration will be performed according to SomaLogic’s standardized pipeline. Protein abundance will be expressed as normalized relative fluorescence units (RFU) and log2-transformed as appropriate for downstream statistical analyses. Residual batch effects will be evaluated and corrected, when necessary, using established statistical methods. Active MMP1 concentrations will be quantified independently using a Human Active MMP1 ELISA kit to obtain absolute protein concentrations. ELISA measurements will be performed in technical triplicate and quantified using standard curve-based interpolation. SomaScan data will be accepted only if all platform-specific quality-control metrics meet SomaLogic acceptance criteria; samples or analytes failing quality-control thresholds will be excluded from downstream analyses. For the Human Active MMP1 ELISA, the lower limits of detection and quantification, the standard-curve range, and intra- and inter-assay coefficients of variation will be reported. Measurements below the lower limit of quantification will be treated as left-censored values using predefined statistical procedures.

### Transcriptomic profiling of BMECs

RNA sequencing will be performed to identify differentially expressed genes (DEGs) in BMECs derived from ECP and HC iPSC lines (**Figure 3D**). Total RNA will be isolated from BMECs on day 4 of differentiation, corresponding to the time point at which the most robust TEER values are observed. RNA-seq libraries will be generated using the Illumina RiboZero TruSeq Stranded Total RNA Library Prep Kit. Sequencing will be performed using the Illumina NovaSeq 6000 platform in a 100-nucleotide paired-end configuration, targeting an average depth of 60 million reads per sample. Reads will be trimmed and aligned to the human reference genome (hg38, UCSC assembly) using standard alignment tools. Differential expression analysis will be performed with a false discovery rate (FDR) threshold of 0.05. Gene ontology enrichment and gene set enrichment analyses will be conducted to characterize biological processes and molecular pathways associated with differential gene expression.

### Transcriptomic profiling of cortical neurons exposed to BMEC-CM

Cortical neurons at day 40 of differentiation will be exposed to BMEC-CM for 5 days prior to RNA isolation. RNA-seq experiments will be performed, as previously described (66), to identify gene expression changes induced by BMEC-secreted factors (**Figure 3E**). Matching conditions will include HC neurons exposed to HC BMEC-CM and ECP neurons exposed to ECP BMEC-CM. Cross-culture conditions will include HC neurons exposed to ECP BMEC-CM and ECP neurons exposed to HC BMEC-CM. Differential expression analyses will be conducted to compare gene expression profiles across matching and cross-culture conditions to delineate the relative contributions of intrinsic neuronal vulnerability and extrinsic BMEC-derived factors to disease-related transcriptional changes.

### Pharmacological inhibition of MMP1 in iPSC-derived BMECs

Pharmacological inhibition of MMP1(e.g., piroxicam and infliximab) will be performed in BMECs derived from ECP iPSC lines to assess effects on barrier integrity (**Figure 4A**). Initial dose-finding experiments will be conducted in a subset of ECP lines (n=2-4) to identify concentrations that optimize barrier rescue without compromising cell viability. Based on these preliminary studies, a single optimized concentration and exposure duration will be selected for subsequent experiments across the full study cohort. Barrier integrity will be monitored continuously using impedance-based measurements on the Axion Maestro Edge platform. Permeability assays and tight junction protein analyses will be performed to corroborate impedance findings. Dose-optimization studies will evaluate multiple concentrations spanning the reported biologically active range for each pharmacological agent, together with exposure durations of approximately 24–72 h, using matched vehicle-treated controls. Cell viability will be assessed in parallel to ensure that observed effects reflect barrier restoration rather than nonspecific cytotoxicity. To evaluate molecular mechanisms, phosphorylation of claudin-5 (CLDN5 T207) and occludin (OCLN T382 and S507) will be quantified using validated phospho-specific antibodies. BMECs treated with MMP1 inhibitors will subsequently be used in functional assays and BMEC-neuron CM experiments to assess downstream effects on neuronal morphology and activity.

**Figure 4:**
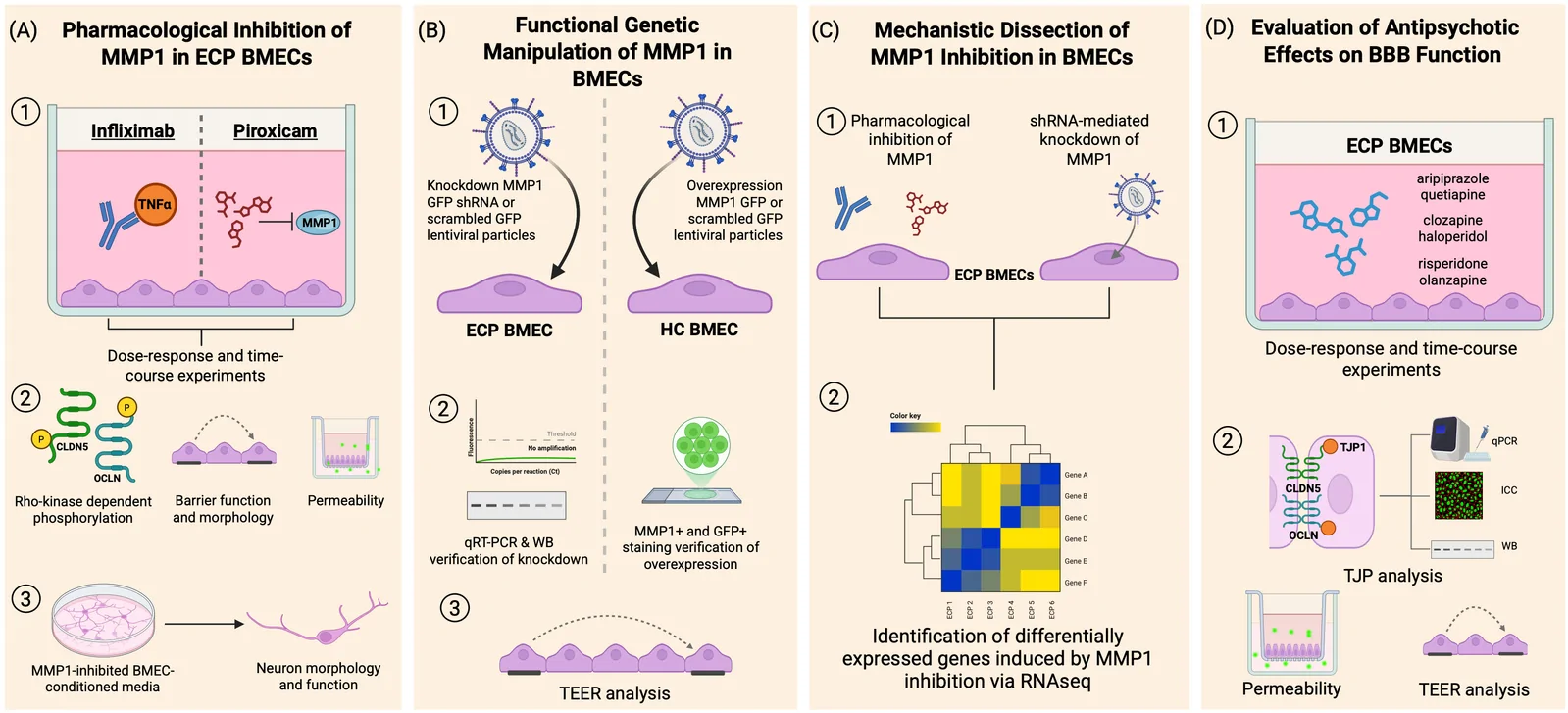
Genetic and pharmacological perturbation of BMECs. **(A)** Pharmacological inhibition of MMP1-related pathways is performed in ECP-derived BMECs using selected compounds (e.g., infliximab, piroxicam), followed by assessment of barrier function and neuronal effects using conditioned media. **(B)** Lentiviral-mediated knockdown and overexpression of MMP1 are performed in ECP and HC BMECs, respectively, to evaluate effects on barrier integrity. **(C)** Transcriptomic profiling is conducted following pharmacological and genetic perturbations to identify molecular mechanisms associated with MMP1 modulation. **(D)** ECP-derived BMECs are exposed to a panel of antipsychotic medications to evaluate their effects on barrier function.

### Functional genetic manipulation of MMP1 in iPSC-derived BMECs

To complement pharmacological inhibition studies, functional genetic manipulation of MMP1 will be performed in iPSC-derived BMECs (**Figure 4B**). For knockdown experiments, MMP1-targeting shRNA lentiviral particles and scrambled controls will be transduced into BMECs derived from ECP lines. Initial optimization experiments will be conducted in a subset of lines to determine multiplicity of infection (MOI) that achieves maximal knockdown efficiency without compromising cell viability. Knockdown efficiency will be quantified by qRT-PCR and immunoblotting, and functional effects will be assessed using impedance-based barrier measurements on the Axion Maestro Edge platform to identify the level of MMP1 suppression associated with maximal barrier rescue.

For overexpression studies, tagged MMP1 lentiviral particles will be transduced into BMECs derived from HC lines, with expression verified by immunostaining and immunoblotting. Following genetic manipulation, barrier integrity will be continuously monitored using impedance-based measurements and corroborated by permeability assays and tight junction protein analyses to determine the impact of altered MMP1 expression on endothelial barrier function.

### Mechanistic dissection of MMP1 inhibition in iPSC-derived BMECs

To represent molecular mechanisms underlying MMP1 inhibition in ECP-derived BMECs, transcriptomic profiling will be performed following pharmacological and genetic perturbations (**Figure 4C**). RNA-seq will be conducted in BMECs treated with infliximab and piroxicam at doses selected based on optimal rescue of barrier function observed in dose-response and time-course experiments. In parallel, RNA-seq will be performed in BMECs following shRNA-mediated knockdown of MMP1. Differential gene expression analyses will be carried out to identify genes and pathways modulated by pharmacological inhibition and genetic suppression of MMP1. Comparative analyses will be performed across conditions to delineate shared and distinct transcriptional signatures associated with MMP1 perturbation. Genes and pathways consistently modulated by both pharmacological and genetic perturbations will be prioritized to characterize molecular mechanisms associated with rescue of BBB function in ECP-derived BMECs.

### Evaluation of antipsychotic effects on BBB function

To evaluate the effects of antipsychotic medications on BBB function, BMECs derived from ECP iPSC lines will be exposed to a panel of antipsychotics in dose-response and time-course experiments (**Figure 4D**). The panel will include aripiprazole and quetiapine (predicted to improve barrier function), clozapine and haloperidol (predicted to destabilize it), risperidone and olanzapine (predicted to have little effect), and other potentially relevant medications. Guided by published literature and preliminary dose-optimization, test concentrations will span clinically relevant plasma ranges (typically low-nanomolar to low-micromolar) with exposure durations of approximately 24–72 h, each compared against matched vehicle-treated controls at equivalent final solvent concentrations. Following treatment, barrier integrity will be assessed by impedance (TEER), permeability assays, and tight junction protein and morphological analyses, with parallel cell-viability assessment to exclude nonspecific cytotoxicity. These are *in vitro* exposures applied directly to BMEC cultures; antipsychotics will not be administered to human participants, and the protocol does not include any clinical drug intervention.

### Statistical analysis

All quantitative analyses will be performed using R and GraphPad Prism. Data will be summarized using descriptive statistics and are reported as mean ± standard deviation (SD) unless otherwise specified. All statistical tests will be two-tailed, with significance defined as *p* < 0.05. Adjustment for multiple comparisons (e.g. false-discovery-rate correction) (67) will be applied where appropriate. To control for potential confounding, various covariates such as age, sex, quality control measures, antipsychotic exposure, cigarette smoking history, diagnosis, and metabolic risk factors (e.g., body mass index) will be included in the relevant analyses. When analyzing longitudinal data, linear mixed-effects models will be used which handle missing data by using available observations at each timepoint.

For BBB assays, including TEER, permeability, efflux transporter activity, and tight junction protein expression, analyses will account for technical replicates and donor-level variability. Measurements will be averaged at the donor level prior to group comparisons unless otherwise specified. Group-level comparisons between ECP- and HC-derived BMECs will be performed using linear mixed-effects models, with diagnostic group and experimental condition as fixed effects and donor identity as a random effect. Batch effects will also be included in these models. For multi-condition experiments (e.g., dose-response or time-course studies), one-way or two-way ANOVA followed by Tukey’s *post hoc* tests will be used as appropriate.

Across all cellular assays, the hierarchical experimental structure (technical replicates nested within plates, plates within differentiation batches, differentiation batches within iPSC clones, and clones within donors) will be explicitly accounted for during statistical analyses. Mixed-effects models will include random intercepts for donor and, where applicable, additional nested random effects for clone and differentiation batch. Technical replicates will generally be averaged before analysis unless variance-component estimation justifies explicit modeling.

Neuronal morphology, synaptic feature quantification, and MEA recordings will be analyzed at the culture level, with values averaged across technical replicates. Comparisons across matching and cross-culture conditions will be performed using linear models or mixed-effects models, with neuronal and BMEC donors included as random effects when applicable.

Inflammatory proteins will be quantified using the SOMAscan inflammation/immune and cytokines response panel and reported as relative fluorescence units (RFU). Raw RFU values will be processed using the standard SOMAscan normalization pipeline, including hybridization normalization, median signal normalization, and calibration across runs. Proteins with more than 25% missing or low-quality measurements will be excluded from downstream analysis. For proteins with fewer than 25% missing values, missing measurements will be imputed using the mean RFU for that analyte across samples. Normalized RFU values will be log2-transformed prior to statistical analysis. Differential protein abundance between groups will be assessed using linear models implemented in the LIMMA package in R. To account for multiple comparisons across proteins, p-values will be adjusted using the Benjamini-Hochberg false discovery rate method, with an adjusted p-value < 0.05 considered statistically significant. Fold changes between groups will be calculated and visualized using volcano plots generated with ggplot2 in R. Functional enrichment analyses of significantly altered inflammatory proteins will be performed using FUNRICH, and protein-protein interaction networks will be explored using STRING (version 11.5) to identify key biological pathways and interaction clusters.

RNA sequencing data will be processed using established pipelines for quality control, normalization, and differential expression analysis. Differential expression testing will be performed using linear modeling approaches with FDR correction set at 0.05. Gene ontology and pathway enrichment analyses will be conducted using standard enrichment frameworks.

Associations between *ex vivo* BBB phenotypes and longitudinal clinical, cognitive, and functional measures will be explored using linear mixed effect models, which will test if BBB function predicts clinical trajectories. Fixed effects will be used for time, the BBB phenotypes, group (ECP vs. HC), and their interactions. Random intercepts and slopes will be used for the clinical, cognitive, and functional data to account for individual variation in the symptoms at baseline and change over time. Relevant covariates will be included in these models too. Clinical data will include different measures of symptoms and clinical severity, while our cognitive data will provide indices of global intelligence as well as specific domains of cognitive processing. Functioning measures will include social, occupational, and global functioning ratings. Lastly, we will test methods of generating composite measures of BBB function and phenotypes and use this composite score for our previous analyses (mixed-effects models and subgrouping) to understand if a multivariate approach to understanding BBB integrity captures relations that individual variables do not. All analysis scripts will be documented and publicly available to support reproducibility.

## Supplemental materials

See **Supplementary Methods** for Inflammatory and physiological stress perturbation experiments, and MMP1 alternative strategies.

## Patient and public involvement

A consumer advisory board at Beth Israel Deaconess Medical Center was consulted for this study. The board consisted of individuals with lived experienced of psychosis and they were engaged in the refinement of the study aims, protocol, and dissemination plan. The recommendations from the board were included in the prospective study protocol.

## Ethics and dissemination

Ethical approval for this study has been obtained from the Beth Israel Deaconess Medical Center. The study will be conducted in accordance with the principles of the Declaration of Helsinki. Written informed consent will be obtained from all participants prior to enrolment. Participant confidentiality will be maintained, and all data will be de-identified and stored securely. Findings from this study will be disseminated through publication in peer-reviewed journals, local mental health events, peer support networks, and presentations at national and international conferences. Clinical trial number: not applicable. No human participant data are reported in the present manuscript, which describes a prospective protocol; the planned study summarized here will involve human participants including recruitment, peripheral blood collection, and longitudinal clinical assessment.

## Discussion

This protocol presents a structured approach for investigating BBB and NVU dysfunction in ECP through the integration of longitudinal clinical characterization with donor-specific *ex vivo* cellular models derived from patient-derived iPSCs. The approach enables systematic assessment of endothelial barrier properties in iPSC-derived BMECs, evaluation of BMEC-driven effects on neuronal structure and function, and interrogation of candidate molecular pathways using targeted pharmacological and genetic perturbations. By linking cellular readouts to repeated clinical, cognitive, and functional measures obtained from the same individuals, the protocol is designed to address biological contributors to clinical heterogeneity and neuroprogression in ECP.

A major limitation in psychiatric studies has been the disconnect between mechanistic cellular studies and longitudinally phenotype patient cohorts (33, 34, 36). Cellular models are often generated independently of clinical trajectories, limiting their relevance to illness course and outcome. The present approach seeks to overcome this gap by embedding donor-derived BBB and NVU phenotypes within a well-characterized longitudinal cohort. This design allows cellular measures to be examined in relation to continuous clinical variables rather than relying solely on categorical diagnostic comparisons. The use of matched and cross-culture paradigms further strengthens interpretability by enabling separation of endothelial-driven effects from intrinsic neuronal characteristics. This is particularly relevant in the context of NVU-based models of psychosis, where vascular, immune, and neuronal processes are increasingly recognized as interacting components rather than independent contributors to disease biology.

Several features enhance the robustness and interpretability of this protocol. First, endothelial barrier function is assessed using paracellular and transcellular assays, including TEER, paracellular permeability, efflux transporter activity, and tight junction protein expression. Together, these measures capture distinct but related aspects of BBB integrity and reduce reliance on any single experimental endpoint. Second, transcriptomic profiling of both BMECs and cortical neurons supports data-driven identification of molecular pathways associated with group differences, clinical trajectories, and experimental perturbations. This unbiased approach facilitates discovery while remaining compatible with hypothesis-driven follow-up studies. Third, pathway interrogation is performed using both pharmacological inhibition and genetic manipulation, allowing mechanistic conclusions to be supported by convergent experimental strategies. Finally, the modular design of the protocol permits iterative expansion as additional subjects, time points, or experimental conditions are incorporated.

CM experiments similarly require consistent collection windows, media formulations, and storage conditions to minimize variability in secreted factor profiles. For neuronal analyses, dendritic and synaptic quantification should be performed using fixed imaging parameters, blinded analysis pipelines, and predefined criteria for neuronal subtype selection. Micro-electrode array recordings are influenced by network maturation and plating density; standardized maturation timelines and analysis windows are therefore critical for reducing non-biological variability.

This protocol employs reductionist *ex vivo* systems and does not fully recapitulate the complexity of the *in vivo* neurovascular environment. Although iPSC-derived BMECs reproduce key features of endothelial barrier function, they do not model shear stress, full NVU cellular diversity, or long-term vascular maturation. CM paradigms provide controlled assessment of secretome-mediated effects but do not capture direct cell-cell contact or contributions from astrocytes, pericytes, or microglia.

Additional variability may arise from iPSC reprogramming and differentiation, including clone-specific effects, passage number, and differentiation efficiency. These factors underscore the importance of multi-clone validation and analytic strategies that account for donor-level clustering. From a clinical perspective, ECP populations are heterogeneous with respect to diagnosis, medication exposure, inflammatory burden, and metabolic risk. These factors may influence both clinical trajectories and endothelial biology and should be addressed through covariate adjustment and sensitivity analyses where possible. Identification of BBB-related subgroups is exploratory and dependent on assay reproducibility and analytic thresholds, necessitating cautious interpretation and independent validation.

This protocol provides a foundation for identifying BBB- and NVU-related biological subtypes within ECP that may not be apparent from clinical measures alone. By relating *ex vivo* cellular phenotypes to longitudinal outcomes, the approach supports nomination of candidate biomarkers and prioritization of molecular pathways for further investigation. The inclusion of pharmacological perturbation modules enables preclinical assessment of pathway-targeted interventions and comparison of antipsychotic effects on endothelial integrity under standardized conditions.

Future extensions may incorporate more complex co-culture systems, flow-based platforms to model vascular shear stress, or immune-endothelial interaction assays. Integration of additional -omic layers, including proteomics, phosphoproteomics and metabolomics, could further refine mechanistic insight. Alignment with *in vivo* measures of BBB permeability, when available, may strengthen translational inference by linking cellular phenotypes to neurovascular alterations observed in patients.

## Conclusion

In summary, this protocol will provide a structured and reproducible approach to model BBB and NVU dysfunction in ECP using integrated longitudinal clinical phenotyping and patient-specific *ex vivo* iPSC-based assays. By enabling parallel assessment of endothelial barrier integrity, BMEC-neuron interactions, transcriptomic signatures, and mechanistic perturbations, the framework supports systematic investigation of cellular and molecular mechanisms that may contribute to heterogeneity and neuroprogression in early course psychosis.

## Data Availability

The original contributions presented in the study are included in the article/**Supplementary Material**. Further inquiries can be directed to the corresponding author.
