# Supplementary material for: Patient-derived stem cell models of brain endothelial-neuron interactions in early course psychosis
Source: Front Psychiatry. 2026 Sep 29;17:1873300. doi: 10.3389/fpsyt.2026.1873300 (PMC13659095; doi:10.3389/fpsyt.2026.1873300)
Supplement: Supplementary file 1 [file Supplementaryfile1.docx]

**Supplemental Methods**

**Inflammatory Stress and Transcriptomic Profiling of iPSC-Derived BMECs**

Intrinsic deficits in iPSC-derived BMECs from individuals with ECP may not be detectable under baseline culture conditions and may instead emerge in response to inflammatory stress. Accordingly, if no significant differences are observed between ECP and HC BMECs under standard culture conditions, a pro-inflammatory priming paradigm will be implemented.

BMECs will be exposed to a panel of pro-inflammatory cytokines, including interleukin-6 (IL-6), tumor necrosis factor alpha (TNFα), interleukin-1 beta (IL-1β), or interferon gamma (IFNγ), to assess whether inflammatory stress unmasks differential barrier phenotypes in ECP-derived BMECs relative to HC-derived BMECs. Following cytokine exposure, BBB integrity will be re-evaluated using TEER, permeability assays, and efflux transporter activity to determine whether ECP BMECs exhibit aberrant functional responses under inflammatory conditions.

If inflammatory priming reveals differential barrier phenotypes between ECP and HC BMECs, transcriptomic profiling will be conducted using RNA sequencing (RNA-seq) to characterize gene expression changes induced by inflammatory stress. RNA-seq analyses will be performed to compare cytokine-stimulated ECP and HC BMECs and to describe molecular pathways mediating differential cellular responses to inflammatory challenge.

**Stress Perturbation and Direct BMEC-Neuron Co-culture Strategies**

ECP-specific neuronal phenotypes may not be detectable under baseline culture conditions and may instead emerge in response to physiological or inflammatory stress. Accordingly, if no significant differences are observed between neurons exposed to ECP vs. HC BMEC-conditioned media under standard culture conditions, experiments will be repeated under defined perturbation conditions. These perturbations will include exposure to pro-inflammatory cytokines, corticosteroids, low-glucose media, or KCl-mediated depolarization to determine whether stress conditions unmask disease-specific neuronal responses^66,68,69^.

Factors secreted by BMECs may influence neuronal structure and function, and conditioned media from ECP BMECs may exacerbate disease-related neuronal phenotypes. However, it is possible that certain effects require direct cellular interaction between BMECs and neurons. Therefore, if BMEC-conditioned media does not produce significant neuronal effects, BMECs and cortical neurons will be co-cultured in the same well to evaluate potential contact-dependent mechanisms.

**Alternative Strategies of MMP1**

Intrinsic variability in MMP1-related barrier phenotypes across ECP-derived BMEC lines may limit the extent to which MMP1 inhibition accounts for BBB dysfunction in all lines. If pharmacological and genetic perturbation experiments indicate that MMP1 does not play a central role in mediating barrier dysfunction in a subset of ECP-derived BMECs, additional candidate proteins exhibiting differential profiles in ECP-derived BMECs will be interrogated, including MMP2, MMP9, CXCL8, CCL2, and CCL19. Transcriptomic analyses may not fully capture proximal signaling events mediating the effects of MMP1 inhibition on BBB integrity. If RNA-seq data do not reveal informative gene expression changes associated with barrier rescue, phosphoproteomic profiling will be performed to define the signaling pathways mediating the effects of MMP1 inhibition in ECP-derived BMECs.

**References**

1.	Griffiths, S. L., Lalousis, P. A., Wood, S. J. & Upthegrove, R. Heterogeneity in treatment outcomes and incomplete recovery in first episode psychosis: does one size fit all? *Transl. Psychiatry* **12**, 485 (2022).

2.	Oomen, P. P. *et al.* Longitudinal clinical and functional outcome in distinct cognitive subgroups of first-episode psychosis: a cluster analysis. *Psychol. Med.* **53**, 2317–2327 (2023).

3.	Lewandowski, K. E., Bouix, S., Ongur, D. & Shenton, M. E. Neuroprogression across the early course of psychosis. *J. Psychiatry Brain Sci.* **5**, e200002 (2020).

4.	Molho, W. *et al.* Retinal electrophysiological alterations are associated with cognition in early course psychosis. *Schizophr. Res.* **274**, 137–141 (2024).

5.	Velez-Perez, E. *et al.* Electroretinographic dysfunction, insulin resistance, and childhood trauma in early-course psychosis: a case-control exploratory study. *Biomark. Neuropsychiatry* **10**, 100088 (2024).

6.	Gong, Y. *et al.* Research developments in the neurovascular unit and the blood‑brain barrier (Review). *Biomed. Rep.* **22**, 1–14 (2025).

7.	Howes, O., Marcinkowska, J., Turkheimer, F. E. & Carr, R. Synaptic changes in psychiatric and neurological disorders: state-of-the art of in vivo imaging. *Neuropsychopharmacology* **50**, 164–183 (2025).

8.	Hall, J. & Bray, N. J. Schizophrenia genomics: convergence on synaptic development, adult synaptic plasticity, or both? *Biol. Psychiatry* **91**, 709–717 (2022).

9.	Howes, O. D. & Onwordi, E. C. The synaptic hypothesis of schizophrenia version III: a master mechanism. *Mol. Psychiatry* **28**, 1843–1856 (2023).

10.	Pong, S., Karmacharya, R., Sofman, M., Bishop, J. R. & Lizano, P. The role of brain microvascular endothelial cell and blood-brain barrier dysfunction in schizophrenia. *Complex Psychiatry* **6**, 30–46 (2020).

11.	Jouan, L. *et al.* Investigation of rare variants in LRP1, KPNA1, ALS2CL and ZNF480 genes in schizophrenia patients reflects genetic heterogeneity of the disease. *Behav. Brain Funct.* **9**, 9 (2013).

12.	Mealer, R. G. *et al.* The schizophrenia-associated variant in SLC39A8 alters protein glycosylation in the mouse brain. *Mol. Psychiatry* **27**, 1405–1415 (2022).

13.	Martinez, M. E., Cox, D. F., Youth, B. P. & Hernandez, A. Genomic imprinting of DIO3, a candidate gene for the syndrome associated with human uniparental disomy of chromosome 14. *Eur. J. Hum. Genet.* **24**, 1617–1621 (2016).

14.	Crockett, A. M. *et al.* Disruption of the blood–brain barrier in 22q11. 2 deletion syndrome. *Brain* **144**, 1351–1360 (2021).

15.	Nishiura, K. *et al.* PKA activation and endothelial claudin-5 breakdown in the schizophrenic prefrontal cortex. *Oncotarget* **8**, 93382 (2017).

16.	Puvogel, S., Palma, V. & Sommer, I. E. Brain vasculature disturbance in schizophrenia. *Curr. Opin. Psychiatry* **35**, 146–156 (2022).

17.	Zhang, F. *et al.* Blood–Brain Barrier Disruption in Schizophrenia: Insights, Mechanisms, and Future Directions. *Int. J. Mol. Sci.* **26**, 873 (2025).

18.	Lindholm Carlström, E. *et al.* Transcriptome analysis of post-mortem brain tissue reveals up-regulation of the complement cascade in a subgroup of schizophrenia patients. *Genes* **12**, 1242 (2021).

19.	North, H. F., Lim, J., Fullerton, J. M., Webster, M. J. & Shannon Weickert, C. Altered Vascular and Angiogenic Transcripts in the Neurogenic Niche in Neuroinflammatory-Type Schizophrenia. *Schizophr. Bull.* sbaf177 (2025).

20.	Puvogel, S. *et al.* Single-nucleus RNA sequencing of midbrain blood-brain barrier cells in schizophrenia reveals subtle transcriptional changes with overall preservation of cellular proportions and phenotypes. *Mol. Psychiatry* **27**, 4731–4740 (2022).

21.	Chukaew, P. *et al.* Correlation of BDNF , VEGF , TNF ‐α, and S100B with cognitive impairments in chronic, medicated schizophrenia patients. *Neuropsychopharmacol. Rep.* **42**, 281–287 (2022).

22.	Meixensberger, S. *et al.* Upregulation of sICAM-1 and sVCAM-1 levels in the cerebrospinal fluid of patients with schizophrenia spectrum disorders. *Diagnostics* **11**, 1134 (2021).

23.	Steiner, J., Bielau, H., Bernstein, H. G., Bogerts, B. & Wunderlich, M. T. Increased cerebrospinal fluid and serum levels of S100B in first-onset schizophrenia are not related to a degenerative release of glial fibrillar acidic protein, myelin basic protein and neurone-specific enolase from glia or neurones. *J. Neurol. Neurosurg. Psychiatry* **77**, 1284–1287 (2006).

24.	Radu, G. *et al.* The Predictive Value of Endothelial Inflammatory Markers in the Onset of Schizophrenia. *Neuropsychiatr. Dis. Treat.* **Volume 16**, 545–555 (2020).

25.	Bai, Y.-L. *et al.* Cell-free DNA in extracellular vesicles: A candidate biomarker of schizophrenia. *World J. Psychiatry* **15**, 107404 (2025).

26.	Perdikakis, M. *et al.* Diagnostic role of circulating cell-free DNA in schizophrenia and neuro-degenerative disorders. *Biomark. Med.* **19**, 165–176 (2025).

27.	Lubotzky, A. *et al.* Elevated brain-derived cell-free DNA among patients with first psychotic episode–a proof-of-concept study. *Elife* **11**, e76391 (2022).

28.	Oviedo-Salcedo, T. *et al.* Cerebrospinal fluid abnormalities in first-and multi-episode schizophrenia-spectrum disorders: impact of clinical and demographical variables. *Transl. Psychiatry* **11**, 621 (2021).

29.	Ricci, F. *et al.* Effect of Vascular Risk Factors on Blood-Brain Barrier and Cerebrospinal Fluid Biomarkers Along the Alzheimer’s Disease Continuum: A Retrospective Observational Study. *J. Alzheimers Dis.* **97**, 599–607 (2024).

30.	Zhao, N. O. *et al.* Blood-brain barrier dysfunction in bipolar disorder: molecular mechanisms and clinical implications. *Brain Behav. Immun.-Health* **21**, 100441 (2022).

31.	Kamintsky, L. *et al.* Blood-brain barrier imaging as a potential biomarker for bipolar disorder progression. *NeuroImage Clin.* **26**, 102049 (2020).

32.	Moussiopoulou, J. *et al.* Higher blood–brain barrier leakage in schizophrenia-spectrum disorders: A comparative dynamic contrast-enhanced magnetic resonance imaging study with healthy controls. *Brain. Behav. Immun.* **128**, 256–265 (2025).

33.	Karmacharya, R., Kieling, C. & Mondelli, V. Integrating stem cell-based experiments in clinical research. *Eur. Psychiatry* **63**, e62 (2020).

34.	Lizano, P. & Karmacharya, R. Harnessing stem cell-based approaches for clinically meaningful discoveries in schizophrenia. *Schizophr. Res.* **273**, 1 (2024).

35.	Watmuff, B., Liu, B. & Karmacharya, R. Stem Cell-Derived Neurons in the Development of Targeted Treatment for Schizophrenia and Bipolar Disorder. *Pharmacogenomics* **18**, 471–479 (2017).

36.	Karmacharya, R. & Haggarty, S. J. Stem cell models of neuropsychiatric disorders. *Molecular and cellular neurosciences* vol. 73 1–2 (2016).

37.	Qian, T. *et al.* Directed differentiation of human pluripotent stem cells to blood-brain barrier endothelial cells. *Sci. Adv.* **3**, e1701679 (2017).

38.	Stebbins, M. J. *et al.* Differentiation and characterization of human pluripotent stem cell-derived brain microvascular endothelial cells. *Methods* **101**, 93–102 (2016).

39.	Vlahos, K. *et al.* Generation of iPSC lines from peripheral blood mononuclear cells from 5 healthy adults. *Stem Cell Res.* **34**, 101380 (2019).

40.	Linville, R. M., Arevalo, D., Maressa, J. C., Zhao, N. & Searson, P. C. Three-dimensional induced pluripotent stem-cell models of human brain angiogenesis. *Microvasc. Res.* **132**, 104042 (2020).

41.	Lippmann, E. S. *et al.* Derivation of blood-brain barrier endothelial cells from human pluripotent stem cells. *Nat. Biotechnol.* **30**, 783–791 (2012).

42.	Lizano, P., Pong, S., Santarriaga, S., Bannai, D. & Karmacharya, R. Brain microvascular endothelial cells and blood-brain barrier dysfunction in psychotic disorders. *Mol. Psychiatry* **28**, 3698–3708 (2023).

43.	Casas, B. S. *et al.* Schizophrenia-derived hiPSC brain microvascular endothelial cells show impairments in angiogenesis and blood-brain barrier function. *bioRxiv* 2022–04 (2022).

44.	Stankovic, I. *et al.* Schizophrenia endothelial cells exhibit higher permeability and altered angiogenesis patterns in patient-derived organoids. *Transl. Psychiatry* **14**, 53 (2024).

45.	Wen, Z., Christian, K. M., Song, H. & Ming, G. Modeling psychiatric disorders with patient-derived iPSCs. *Curr. Opin. Neurobiol.* **36**, 118–127 (2016).

46.	Kathuria, A. *et al.* Synaptic deficits in iPSC-derived cortical interneurons in schizophrenia are mediated by NLGN2 and rescued by N-acetylcysteine. *Transl. Psychiatry* **9**, 321 (2019).

47.	Santarriaga, S., Gerlovin, K., Layadi, Y. & Karmacharya, R. Human stem cell-based models to study synaptic dysfunction and cognition in schizophrenia: A narrative review. *Schizophr. Res.* **273**, 78–97 (2024).

48.	Aryal, S. *et al.* Deep proteomics identifies shared molecular pathway alterations in synapses of patients with schizophrenia and bipolar disorder and mouse model. *Cell Rep.* **42**, (2023).

49.	Kathuria, A., Lopez-Lengowski, K., Watmuff, B. & Karmacharya, R. Morphological and transcriptomic analyses of stem cell-derived cortical neurons reveal mechanisms underlying synaptic dysfunction in schizophrenia. *Genome Med.* **15**, 58 (2023).

50.	Gonzalez-Burgos, G., Miyamae, T., Nishihata, Y., Krimer, O. L. & Lewis, D. A. Strength of excitatory inputs to layer 3 pyramidal neurons during synaptic pruning in the monkey prefrontal cortex: Relevance for the pathogenesis of schizophrenia. *Biol. Psychiatry* **94**, 288–296 (2023).

51.	Kathuria, A. *et al.* Transcriptome analysis and functional characterization of cerebral organoids in bipolar disorder. *Genome Med.* **12**, 34 (2020).

52.	Kathuria, A., Lopez-Lengowski, K., McPhie, D., Cohen, B. M. & Karmacharya, R. Disease-specific differences in gene expression, mitochondrial function and mitochondria-endoplasmic reticulum interactions in iPSC-derived cerebral organoids and cortical neurons in schizophrenia and bipolar disorder. *Discov. Ment. Health* **3**, 8 (2023).

53.	Kathuria, A. *et al.* Transcriptomic landscape and functional characterization of induced pluripotent stem cell–derived cerebral organoids in schizophrenia. *JAMA Psychiatry* **77**, (2020).

54.	Kempuraj, D. *et al.* Neurovascular unit, neuroinflammation and neurodegeneration markers in brain disorders. *Front. Cell. Neurosci.* **18**, 1491952 (2024).

55.	Reyes, R. *et al.* Role of tumor necrosis factor–α and matrix metalloproteinase–9 in blood-brain barrier disruption after peripheral thermal injury in rats. *J. Neurosurg.* **110**, 1218–1226 (2009).

56.	Leib, S. L., Leppert, D., Clements, J. & Täuber, M. G. Matrix Metalloproteinases Contribute to Brain Damage in Experimental Pneumococcal Meningitis. *Infect. Immun.* **68**, 615–620 (2000).

57.	Rempe, R. G. *et al.* Matrix metalloproteinase-mediated blood-brain barrier dysfunction in epilepsy. *J. Neurosci.* **38**, 4301–4315 (2018).

58.	Yamamoto, M. *et al.* Phosphorylation of claudin-5 and occludin by rho kinase in brain endothelial cells. *Am. J. Pathol.* **172**, 521–533 (2008).

59.	Hollmann, E. K. *et al.* Accelerated differentiation of human induced pluripotent stem cells to blood–brain barrier endothelial cells. *Fluids Barriers CNS* **14**, 9 (2017).

60.	Neal, E. H. *et al.* A simplified, fully defined differentiation scheme for producing blood-brain barrier endothelial cells from human iPSCs. *Stem Cell Rep.* **12**, 1380–1388 (2019).

61.	Faul, F., Erdfelder, E., Lang, A.-G. & Buchner, A. G*Power 3: A flexible statistical power analysis program for the social, behavioral, and biomedical sciences. *Behav. Res. Methods* **39**, 175–191 (2007).

62.	Faul, F., Erdfelder, E., Buchner, A. & Lang, A.-G. Statistical power analyses using G*Power 3.1: Tests for correlation and regression analyses. *Behav. Res. Methods* **41**, 1149–1160 (2009).

63.	Lu, T. M. *et al.* Pluripotent stem cell-derived epithelium misidentified as brain microvascular endothelium requires ETS factors to acquire vascular fate. *Proc. Natl. Acad. Sci.* **118**, e2016950118 (2021).

64.	Chambers, S. M. *et al.* Highly efficient neural conversion of human ES and iPS cells by dual inhibition of SMAD signaling. *Nat. Biotechnol.* **27**, 275–280 (2009).

65.	Borghese, L. *et al.* Inhibition of notch signaling in human embryonic stem cell–derived neural stem cells delays G1/S phase transition and accelerates neuronal differentiation in vitro and in vivo. *Stem Cells* **28**, 955–964 (2010).

66.	Kathuria, A., Lopez-Lengowski, K., Roffman, J. L. & Karmacharya, R. Distinct effects of interleukin-6 and interferon-γ on differentiating human cortical neurons. *Brain. Behav. Immun.* **103**, 97–108 (2022).

67.	Benjamini, Y. & Hochberg, Y. Controlling the False Discovery Rate: A Practical and Powerful Approach to Multiple Testing. *J. R. Stat. Soc. Ser. B Stat. Methodol.* **57**, 289–300 (1995).

68.	Huang, J. H. *et al.* Perturbational profiling of metabolites in patient fibroblasts implicates α-aminoadipate as a potential biomarker for bipolar disorder. *Complex Psychiatry* **2**, 97–106 (2016).

69.	Huang, J. H. *et al.* Unbiased Metabolite Profiling of Schizophrenia Fibroblasts under Stressful Perturbations Reveals Dysregulation of Plasmalogens and Phosphatidylcholines. *J. Proteome Res.* **16**, 481–493 (2017).
